# Diurnal changes in postural control in normal children: Computerized static and dynamic assessments

**DOI:** 10.4103/2321-3868.136826

**Published:** 2014-07-28

**Authors:** Sophie Bourelle, Redha Taiar, Benoit Berge, Vincent Gautheron, Jerome Cottalorda

**Affiliations:** 1 Department of Pediatric Surgery, University Hospital of Reims, France; 2 Department of Sport Sciences, University of Reims Champaign, France; 3 Euraxi Pharma, Joué-Lès-Tours, France; 4 Physical and Rehabilitation Medicine Service, University Hospital Saint-Etienne, France; 5 Department of Orthopaedic Pediatric and Plastic Surgery, University Hospital Montpellier, France

**Keywords:** Child, postural control, posturography, diurnal patterns, trauma prevention

## Abstract

Mild traumatic brain injury (mTBI) causes postural control deficits and accordingly comparison of aberrant postural control against normal postural control may help diagnose mTBI. However, in the current literature, little is known regarding the normal pattern of postural control in young children. This study was therefore conducted as an effort to fill this knowledge gap. Eight normal school-aged children participated. Posture assessment was conducted before (7–8 a.m. in the morning) and after (4–7 p.m. in the afternoon) school on regular school days using the Balance Master® evaluation system composed of 3 static tests and 2 dynamic balance tests. A significant difference in the weight-bearing squats was detected between morning hours and afternoon hours (*P* < 0.05). By end of afternoon, the body weight was borne mainly on the left side with the knee fully extended and at various degrees of knee flexion. A significantly better directional control of the lateral rhythmic weight shifts was observed at the end of the afternoon than at morning hours (*P* < 0.05). In summary, most of our findings are inconsistent with results from previous studies in adults, suggesting age-related differences in posture control in humans. On a regular school day, the capacity of postural control and laterality or medio-lateral balance in children varies between morning and afternoon hours. We suggest that posturographic assessment in children, either in normal (e.g., physical education and sports training) or in abnormal conditions (e.g., mTBI-associated balance disorders), be better performed late in the afternoon.

## Introduction

Our health and wellbeing are influenced by a wide range of factors, including socio-economic status, cultural background, psychological health and environmental hazards. These factors are changing dynamically as we progress through the key transition points in life, from infancy and childhood, through our teenage years, to adulthood, working life, retirement and the end of life. Along with these transition changes in life, the capacity of postural control that is a prerequisite for performing routine physical activities such as walking, going up stairs, picking up an object from the floor or catching up yourself when the bus is coming to a sudden halt is also changing.

**Table:** 

Access this article online	
**Quick Response Code**:	**Website**: www.burnstrauma.com
	**DOI**: 10.4103/2321-3868.136826

Adequate postural control is defined as the capacity to maintain or to replace the center of gravity within the surface of a support basis. It has been documented that normal postural control largely relies on the integrated information of the vestibular, visual and somatosensorial systems[1,2] and that mild traumatic brain injury (mTBI) may seriously impair postural control.[3] Given the demonstrated relationship between postural control and physiopathology of the brain, assessment of aberrant postural control against normal postural control may be of a significant clinical importance in the diagnosis of brain lesions including mTBI. Accordingly, normal postural control in healthy subjects needs to be better understood.

Like many physiological processes, the level of postural control varies with physical, bio-mechanical, metabolical and psychosocial conditions.[4–9] To date, most studies on postural control are conducted in adult subjects, in whom impaired postural control has been demonstrated to be associated with fatigue and lack of sleep;[10,11] postural control in children is relatively less studied. The aim of this work was to assess changes in postural control at various time points of typical school days in normal healthy children.

## Materials and methods

### Subjects

Eight children (5 boys and 3 girls with an average age of 9 years and 4 months) were recruited from a regular public school in Saint-Etienne, France. They were in the same grade with the same workload and a similar degree of physical and mental fatigue throughout a class day. They had no medical history and were sensorial deficit-free with normal comprehension capabilities. They were all right-handed.

### Posturographic analysis

**Figure 1:**
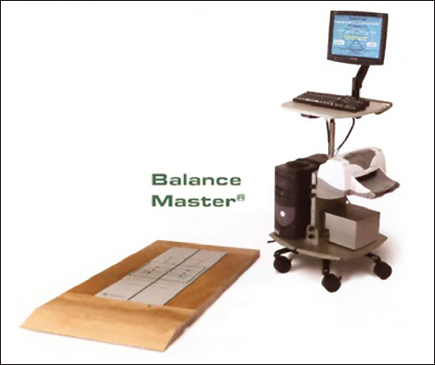
Photo of the Balance Master®.

The postural control was assessed in all subjects before (7–8 a.m.) and after (4–7 p.m.) school on regular school days using the Balance Master® (version 7.0 de NeuroCom International) system.[4,12] As shown in Figure 1, the Balance Master® machine is 152 cm long and 46 cm wide with the central part as the measurement unit, which is connected to a computer. The following 5 specific tests were performed.

#### Weight bearing squat (WBS)

**Figure 2:**
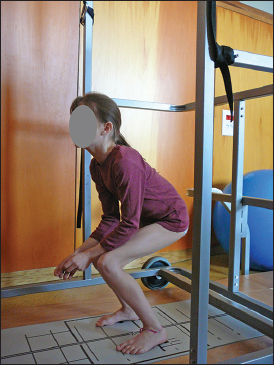
Child doing the test of body weight distribution with bent knee.

The distribution of the body weight in the lower limbs with knees extended and at 30°, 60° and 90° of knee flexion was assessed [Figure 2]. The results were expressed as percentage of the body weight on each leg.

#### Modified clinical test for sensory interaction on balance (mCTSIB)

To determine the oscillation speed of the subject’s center of gravity necessary to maintain body balance while standing on the platform of the Balance Master machine, the mCTSIB was performed. To this end, 3 recordings of 10 seconds each were performed in 4 different conditions: Open eyes on a stable solid ground, open eyes on a foam rubber, closed eyes on a solid ground, and closed eyes on a foam rubber, respectively. The speed of oscillation in each test was expressed as degrees per second. The average of the 3 recordings and the overall average of the measurements under the 4 conditions were automatically calculated.

#### Unilateral Stance (US)

To determine the speed of oscillation of the subject’s center of gravity necessary to maintain body balance on unilateral stance on the platform, US test was performed. Three recordings of 10 seconds each were conducted in 4 different situations (i.e., on the right foot with eyes open and closed respectively; on the left foot with eyes open and closed respectively). The speed of oscillations was expressed as degrees per second.

#### Limits of stability (LOS)

**Figure 3:**
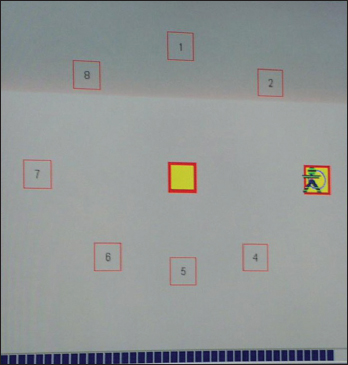
Screen of the computer during the test of stability limits, representing the localization of the peripheral targets and allowing the visual feedback.

To evaluate the capacity of a subject to voluntarily move the center of body gravity in different directions in the limits of theoretical stability (spatial location allowing bending without modifying body stance and briefly standing still in different positions), LOS test was performed. The localization of the subject’s center of gravity was represented on the screen by a cursor allowing a visual feedback. The patient had to lean quickly and accurately from the center rest position to eight peripheral targets set out every 45° and briefly maintain stability at those positions [Figure 3].

In each test, the subject was asked to move his center of gravity to a peripheral target for 8 seconds. The subject was allowed to do pre-test practice needed to achieve a good control of the cursor by the movements of the center of gravity. The position and the movements of the center of gravity were recorded continuously.

In each of the attempts to reach the 8 designated targets, parameters including the response time, the speed of movement, the initial arrival point of movement (Endpoint Excursion, EPE), the final arrival point of movement (Maximum Excursion, MXE) and the directional control of movement were measured. The response time was expressed as the duration in seconds between the movement start signal (appearance of a circle inside of a peripheral target) and the beginning of the actual movement, EPE as the distance in percent of the balance limits, representing the initial attempt to reach a peripheral target, and MXE also as distance in percent of the balance limits, representing the longest distance ran by the center of gravity throughout the test. Accordingly, EPE represented the capacity to anticipate the movement (*feedforward*) and MXE the capacity of correcting the movement (*feedback*). The speed of movement represented the average quickness of movement of the center of gravity, expressed in degrees per second, between 5% and 95% of EPE. The directional control, expressed in percentage, represented the proportion of movement exclusively directed towards the target throughout the shifting of the center of gravity towards the target.

#### Rhythmic weight shifts (RWS)

To evaluate the quality of execution of swinging rhythmic movements of the center of gravity antero-posterior and lateral at 3 different given speeds (3, 2 and 1 second of transition), RWS test was conducted. The center of gravity of the subject was represented on the screen by a cursor allowing a visual feedback. The measured parameters were: The speed of movement on the axis, expressed in degrees per second, and the directional control in the 2 types of shifting and at 3 different speeds.

### Statistical analysis

The Wilcoxon signed-rank test has been done to compare the data of the posturographic tests between morning and afternoon. A two-way repeated-measures analysis of variance after a rank transformation was performed to evaluate statistical differences between posturographic conditions and at morning vs. evening times. The level of significance has been set to 0.05. The calculations have been done with the software SAS version 9.1 by a biostatistician.

## Results

### Body weight distribution

**Figure 4:**
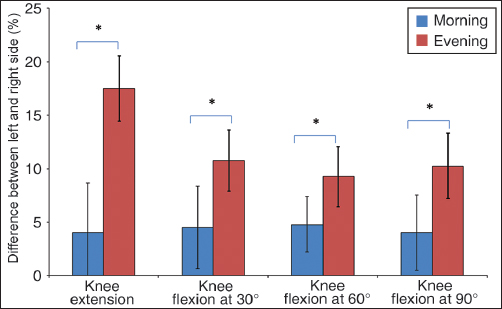
Body weight distribution between left and right side. No significant difference was observed at all degrees of knee flexion at morning (*P > 0.05). Contrary the inverse was observed when the assessment was performed in the afternoon (*P < 0.05).

As shown in Figure 4, there was no significant difference in the distribution of the body weight between left and right legs at all degrees of knee flexion at morning hours (*P* > 0.05). In contrast, the body weight was distributed significantly more on the left side than the right side when the assessment was performed at all degrees of knee flexion at afternoon hours (*P* < 0.05).

### Oscillation speed (mCTSIB)

There was no significant difference in the speed of oscillation measured on a hard or foam rubber floor between morning and afternoon hours (*P* > 0.05). Regardless of whether eyes were open or closed, the speeds of oscillation remained unchanged.

### Unilateral stance

There was no significant difference in the speed of oscillation either on the right side or on the left side between morning hours and afternoon hours (*P* > 0.05). It made no difference whether eyes were open or closed.

### Rhythmic movements

**Figure 5:**
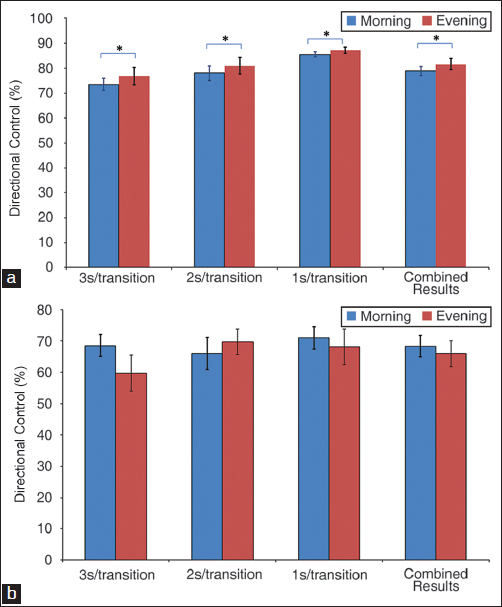
Directional control of children doing the test of the rhythmical balance movements: (a) For the test of the rhythmical balance movements (RWS) (left/right) a significant difference was observed with regard to the lateral swinging movements (*P < 0.05). The subject had a better directional control during the afternoon. (b) For the test of the RWS (forward/back) no significant difference was observed in terms of antero-posterior swing movements between morning hours and afternoon.

With regard to the lateral swinging movements, the subjects had a better directional control at afternoon hours than at morning hours, as is shown in Figure 5a (*P* < 0.05). In terms of anteroposterior swing movements, there was no significant difference in the capacity of directional control between morning hours and afternoon hours, as is shown in Figure 5b (*P* > 0.05).

### Limits of stability

All the parameters [Table 1] assessed in this regard remained relatively consistent; no significant differences were observed between morning hours and afternoon hours (*P* > 0.05).

**Table 1:** **Limits of stability: Synthesis of the composite results**

Limits of stability (composite results)	Response time (s)	Speed of movement (°/s)	EPE (%)	MXE (%)	Directional control (%)
Morning	0.74±0.13	7.14±2.05	75.63±9.61	95±4.75	64.75 ±8.19
Evening	0.75±0.18	6.99±2.52	73.38±12.35	94.25±5.18	65.25±10.39

No significant result was observed, *P* > 0.05 by Wilcoxon test

## Discussion

In this study, we demonstrated that the control of the static and dynamic balance in children was relatively stable at various time points of a typical school day. This piece of information would be of particular importance to future studies or clinical trials on postural control where the optimal timing of assessment needs to be decided.

Numerous physiological functions undergo diurnal variations that obey a circadian rhythm.[13,14] Within a cycle of 24 hours, the metabolic and cognitive processes that intervene in the intellectual and physical activities vary.[15] As a result, individually optimal time windows within a cycle may exist for different aspects of human activities.[15,16] In adult subjects, the strength, the response time, the body temperature and the cardiac beat, in relation with the physical activity, sports performances and the cognitive capabilities, are all at the optimal level in the afternoon up to early evening.[13,16]

In this study, the static postural control on bipedal stance, evaluated by the measurements of the speeds of oscillation by the test mCTSIB on a hard or a foam rubber surface, did not vary significantly between morning and afternoon. Likewise, the influence of eye closing was not significant at morning and afternoon hours either. It can be ventured to hypothesize that within the frame of our work, the fatigue accumulated throughout a day of school has no influence on the quality of proprioception, regardless of the surface nature of the stance, and on the way to handle the absence of visual information.

The results from previous studies on adult subjects are inconsistent. Gribble *et al.* showed that the static postural control was influenced highly by the time of the day (i.e., a better control in the morning and systematically worse control around 6 p.m.) but less by the degree of fatigue related to the lack of sleep.[17] On the contrary, Nakano *et al.* reported that the speed of oscillation of the center of gravity increased at a significantly greater extent way with closed eyes and at the time when the rectal temperature was low (e.g., between 5 a.m. and 8 a.m.), suggesting that low body temperature in the morning may lead to a dysfunction of the muscles essential to the maintenance of body balance.[18]

In this study, we also showed that the unilateral stance balance was relatively stable in children during a typical school day with no significant difference between morning and afternoon. In the study of Gribble *et al.*[15], however, the static postural control, on unilateral stance, was demonstrated to be better in the morning. This observation is not only inconsistent with our finding in this regard but also surprisingly conflicting with these authors’ own data on the time for optimal sports performance in the afternoon and early evening. These authors suggest that a better static postural control on unilateral stance in the morning might be associated with a better cognitive functionality, following a circadian rhythm, thus allowing for a sustained focus in the morning. Nevertheless, both this study and the study of Gribble *et al.*[15] showed that the influence of eye closing was not different between early morning and late afternoon.

In addition to the assessment of static postural control, we also performed the dynamic LOS test that evaluates motor and cognitive capabilities to respond to the complex task to initiate a movement, with certain accuracy, in function of a visual stimulus. We demonstrated that the dynamic postural control was surprisingly stable between morning and afternoon (especially for the combined results), inconsistent with the observation of Gribble *et al.* in this aspect.[15] These authors suggest that opposed to the static postural control, dynamic postural control would require more important muscular contractions to process the requested task and these contractions are stronger in the afternoon.

An inverted proportional relationship between the speed and the accuracy at which a repeated test is done, particularly with the accuracy that would be worse in the early evening, has been proposed.[19,20] To assess this relationship in healthy school children in this study, we performed RWS test. Our results showed that the directional control that represents the accuracy of the movement did not vary significantly between morning and afternoon when the swinging is antero-posterior, even though it is better at afternoon concerning the lateral swing. This observation is against the notion that accuracy performances are better in the morning. We speculate that if the performance accuracy varies during the day (being better in the morning), a sort of compensation would occur through enhancement in physical and motor capabilities or by the effect of learning. In terms of the motor capability, the dorsal flexion of the ankle, is a prime factor of success of antero-posterior swinging movements.[21] However, it seems that the dorsal flexion of the ankle, in the adult, does not vary with the circadian rhythm[20] and would be stable throughout the day instead. In terms of the lateral swinging, the improvement in the directional control might be explained by a possible learning curve which, in an unexplainable manner does not appear in the antero-posterior swing, and also by the significant difference in body weight distributions observed between morning and afternoon. In the morning, there was no predominance in the body weight distribution but was more on the left side in the afternoon, even though the children in this study were all right-handed. This observation is in-line with the results from the study of Matsusaka *et al.* where the left leg dominance was demonstrated in the majority of right-handed children.[22] Described as ‘dominant’, the lower left limb in the children in the study of Matsusak *et al.* was believed to function to stabilize the static balance and to control the medio-lateral balance when walking.[22]

In our study, it is interesting to note that it does not seem to exist a preferential foot of support in the morning, when at night the body weight distribution is done more specifically on the left side. The morning tests have been done very early, between 7 and 8 a.m., shortly after waking up. It can then be asked if the fact of having been in a laying down position during several hours prior does not mask the left or right handedness of the lower limbs. Nothing in the literature has been found on the subject and therefore, it is just an hypothesis. One other aspect is that in the test of RWS, the directional control is getting significantly better for the lateral swing when there is no significant improvement in the antero-posterior movements. This observation seems to support the hypothesis that a reinforcement of the dominant side (in this study the left side for all right-handed children) throughout the day may consequently result in better mediolateral balance capabilities.

One limitation of this study is associated with the concern over the reproducibility of the measurements on the Balance Master® in children. Another limitation is the small sample size used.

In summary, for the first time in this study, we have characterized diurnal changes in postural control in healthy school-aged children through computerized static and dynamic assessments and have demonstrated that the postural control pattern during a typical school day in children is different from that in adults. Given the causative role for mild brain trauma in postural control impairment, assessment of alterations in postural control against the postural control pattern characterized for health young children in this study would be helpful to the diagnosis of mild brain trauma, which remains a significant clinical challenge. Nevertheless, our findings warrant further validation in more studies, involving the use of a larger sample size and more reproducible assessment methods.
